# Microbiome Crosstalk in Immunotherapy and Antiangiogenesis Therapy

**DOI:** 10.3389/fimmu.2021.747914

**Published:** 2021-10-21

**Authors:** Xueting Wan, Mengyao Song, Aiyun Wang, Yang Zhao, Zhonghong Wei, Yin Lu

**Affiliations:** ^1^Jiangsu Key Laboratory for Pharmacology and Safety Evaluation of Chinese Materia Medica, School of Pharmacy, Nanjing University of Chinese Medicine, Nanjing, China; ^2^Jiangsu Joint International Research Laboratory of Chinese Medicine and Regenerative Medicine, Nanjing, China; ^3^Jiangsu Collaborative Innovation Center of Traditional Chinese Medicine (TCM) Prevention and Treatment of Tumor, Nanjing University of Chinese Medicine, Nanjing, China; ^4^Department of Biochemistry and Molecular Biology, School of Medicine & Holistic Integrative Medicine, Nanjing University of Chinese Medicine, Nanjing, China

**Keywords:** microbiome, immunotherapy, immune checkpoint, angiogenesis, prebiotics, Chinese medicine

## Abstract

The human body and its microbiome constitute a highly delicate system. The gut microbiome participates in the absorption of the host’s nutrients and metabolism, maintains the microcirculation, and modulates the immune response. Increasing evidence shows that gut microbiome dysbiosis in the body not only affects the occurrence and development of tumors but also tumor prognosis and treatment. Microbiome have been implicated in tumor control in patients undergoing anti- angiogenesis therapy and immunotherapy. In cases with unsatisfactory responses to chemotherapy, radiotherapy, and targeted therapy, appropriate adjustment of microbes abundance is considered to enhance the treatment response. Here, we review the current research progress in cancer immunotherapy and anti- angiogenesis therapy, as well as the unlimited potential of their combination, especially focusing on how the interaction between intestinal microbiota and the immune system affects cancer pathogenesis and treatment. In addition, we discuss the effects of microbiota on anti-cancer immune response and anti- angiogenesis therapy, and the potential value of these interactions in promoting further research in this field.

## 1 Introduction

The increasing significance of tumor angiogenesis and tumor immune microenvironment in cancer pathogenesis (1) has resulted in the development of anti-angiogenesis therapy and immunotherapy. Numerous studies have reported complex regulatory interactions between the two therapeutic strategies, with increasing clinical research being performed on the use of a combination of these therapies (2). The combined strategy has shown synergistic efficacy in a variety of tumor types (1, 3, 4). For example, a combination of atezolizumab, a PD-L1 monoclonal antibody, and anti-VEGF bevacizumab increased the overall survival of patients with advanced liver cancer in a phase III trial; the combination was approved by FDA in 2019 (5, 6) (**Table 1**). However, the combination of anti-VEGFR2 and anti-PD-L1 did not improve the survival rate of patients with glioblastoma (7), which could be attributed to the low formation rate of high endothelial venules (HEVs) in these tumors—contrary to the findings in breast cancer and primitive neuroectodermal tumor (PNET) models. HEVs are specialized vascular units present in tertiary lymphoid structures that promote the recruitment and exudation of immature T cells, consequently enhancing cytotoxic T lymphocyte (CTL) differentiation. In addition, the expression of ICAM1 and VCAM1 on HEV-rich endothelial cells (ECs) has been known to promote the homing and migration of immune cells to the tumor (7). The anti-programmed cell death protein 1 (PD-1) therapy alone can only bring survival benefits to a small number of patients with glioblastoma (8) with no improvement in the prognosis as reported by a randomized phase III trial (9). Similarly, both anti-VEGF therapy and immunotherapy are ineffective in highly pro-fibroproliferative tumors, such as pancreatic ductal adenocarcinoma (10), breast cancer, and cholangiocarcinoma, indicating the tumor specificity of the combination therapy (11). The most recent literature reports the incidence of grade 3 immune-related adverse events in 49% of 77 advanced melanoma patients receiving anti-CTLA-4 and anti-PD-1 combined immune checkpoint blocking therapy (ICB) and demonstrates the correlation between intestinal microbiota and the response of ICB. Finally, it was proved that the abundance of Bacteroides in the intestinal tract of patients with toxicity was significantly increased, and Bacteroides were associated with intestinal IL-1β and colitis of patients, and the intestinal flora of tumor-bearing mice could mediate the intestinal toxicity induced by ICB through IL-1β.

**Table 1:** Ongoing phase III clinical trials involving combinations of anti-angiogenic inhibitors and cancer immunotherapeutics.

Anti-Angiogenic target	Anti- tumor immunity targe*t*	Combination drugs	Malignancy		Trails
VEGF-A	PD-L1	Bevacizumab	Advanced renal cell carcinoma		NCT02420821
		+Atezolizumab			
VEGFR-1–3, PDGFRβ,	PD-L1	Axitinib	Advanced renal cell carcinoma		NCT02684006
		+ Avelumab		
VEGF-A	PD-L1	Bevacizumab+	Stage NSCLC IV		NCT02366143
		MPDL3280A			
		+ Carboplatin			
		+ Paclitaxel			
VEGF-A	PD-L1	Bevacizumab+	Recurrent Ovarian, Fallopian tube peritoneal cancer		NCT02839707
		Atezolizumab+ Pegylated Liposomal Doxorubicin Hydrochloride			
VEGFR-2, PDGFRβ,	GM-CSF (virus based vaccine)	Sorafenib	Hepatocellular carcinoma		NCT02562755
		+Pexa Vec			

Lederberg et al. (12) referred to the human microbiome as a general term for all microorganisms, including bacteria, fungi, and viruses, as well as their genetic information and metabolites, present in different parts of the human body (such as the gastrointestinal system, respiratory system, skin, reproductive system, and oral cavity).

The human microbiota, often referred to as the “forgotten organ”, (13) comprises 100 times more metagenomes than the human genome. The microbiota participates in key functions related to human health (14), including physiological (15) activities such as nutrition absorption, vitamin synthesis, exogenous substance metabolism, and immune regulation. An imbalance in their abundance is closely related to infections, autoimmune diseases, obesity, diabetes, cardiovascular diseases, and cancer (16, 17).

Studies have implicated chronic infections as a contributory factor to the development of cancer, with about 18% of the global cancer burden directly attributable to infectious agents (18–20). A breakthrough in cancer research occurred in 2017 when researchers and clinicians reported an intricate link between cancer and commensal bacterial species (21).

These findings were confirmed by studies on sterile animals showing that microbial flora in various organs, including skin (22) colon (23), liver (24), breast, and gastrointestinal tract (25), can promote both hereditary and carcinogenic cancers. Emerging evidence shows crosstalk between the tumor microbiome and the gut microbiome. For example, metabolites released by gut microbiota modulate the immune response to pancreatic cancer affecting its progression, immune response, and treatment (26). Metastatic lesions in colorectal cancer are known to be associated with several bacteria including *Fusobacterium* and its associated microbiota, such as *Bacteroides*, *Selenomonas*, and *Prevotella* (27). Epidemiological correlation analysis of colorectal cancer patient cohort and transplantation of patient-derived tumor cells into immunodeficient mice showed that mouse xenografts retained *Fusobacterium* and its associated microbiota. In addition, antibiotic treatment reduced tumor growth in *Fusobacterium*-positive mice. *Clostridium*, a member of the oral microbiota, is associated with colorectal adenoma and adenocarcinoma in human and intestinal tumors in mice (28, 29). In addition, biological disorders and infections caused by excessive use of antibiotics may increase the incidence of certain cancers. Although the use of certain antibiotics has been shown to reduce the incidence of gastric cancer and colorectal cancer, a large-scale epidemiological study (30) conducted in humans reported that exposure to antibiotics may increase the frequency of lung cancer, prostate cancer, and bladder cancer. For instance, excessive use of tetracycline and sulfonamides has been reported as a risk factor for breast cancer.

## 2 Current Classification and Mechanism Of Tumor Immunotherapy

The immune system is the guardian of our body’s integrity, protects the system from foreign invaders including bacteria, viruses, some other pathogens and uncontrolled cancer cells (31). Normally, when tumor cells invade the body’s healthy tissues, the immune system can recognize and eliminate tumor-associated antigens (TAAs) expressed on their surfaces (32). However, tumor cells can inhibit the host immune system through a variety of mechanisms to escape the attack of the immune system. There are four main mechanisms of tumor immune escape: First, the expression of its surface antigen is down- regulated to reduce its immunogenicity, so that it cannot effectively activate the body’s immune system. Second, the expression of immune checkpoints on the cell surface (such as PD-L1) is upregulated to inhibit the activity of T lymphocytes and escape the body’s immune system. Third, immunosuppressive cells [myeloid-derived suppressor cells (MDSCs) and regulatory T cells (Tregs)] are recruited to the tumor immune microenvironment, where they secrete cytokines to evade the immune response of the body to tumor cells. Fourth, acidic and toxic metabolites are released to inhibit the activity of immune cells in the tumor microenvironment (TME).

**Figure 1:**
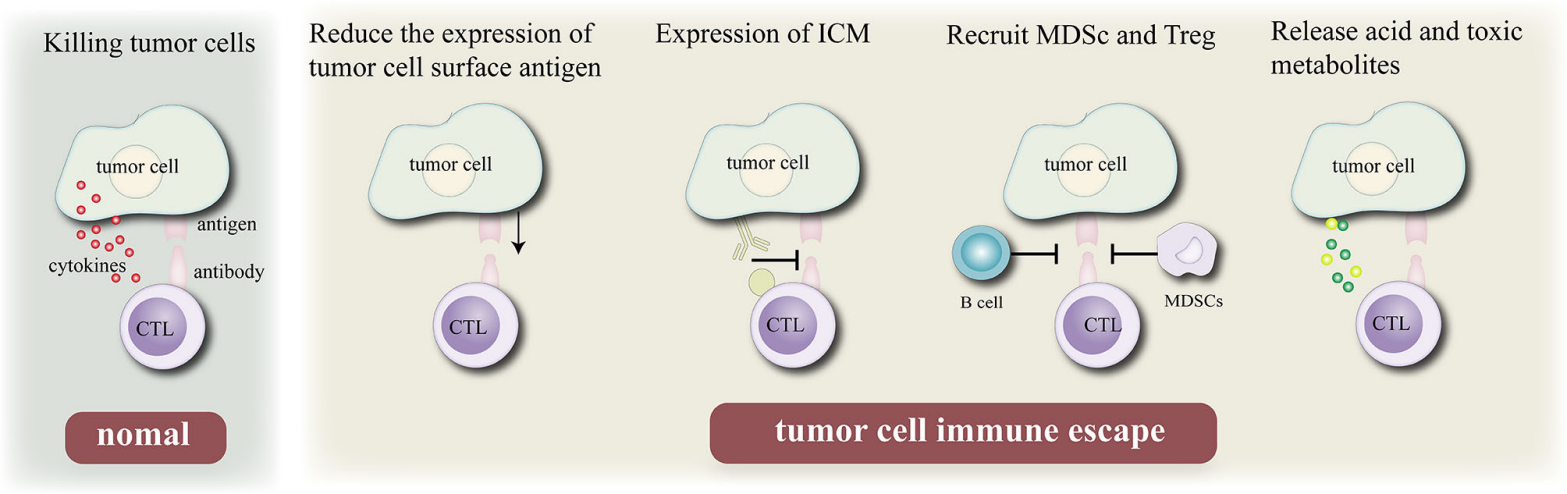
Mechanism of tumor cell immune escape. Normally, cytotoxic T lymphocytes kill tumor cells by recognizing antigenic determinants on the surface of tumor cells and secreting cytokines, Immune escape can be achieved by reduce their immunogenicity by reducing the expression of their own antigens, expressing immune checkpoint molecules, recruiting B cell and MDSC, and releasing acidic or toxic substances to escape the surveillance of the body’s epidemic system.

In recent years, a deep understanding of the mechanism underlying tumor immune escape (33) has resulted in the development of several immunotherapies (**Figure 1**), Since the development of the first tumor immunotherapy drug interferon (IFN)-α, several immunotherapy drugs, such as immune checkpoint (PD-1/PD-L1, CLTA-4, etc.) inhibitors, CAR T cell therapy, tumor vaccine, and oncolytic viruses, have been approved because of their good therapeutic effects. In December 2013, *Science* named tumor immunotherapy as the top 10 scientific and technological breakthroughs of the year, and the 2018 Nobel Prize in physiology/medicine was awarded in this field. Different immunotherapy strategies include cytokine immunotherapy (34); antibody immunotherapy including therapeutic monoclonal antibody (35), immunosuppressive cell MDSCs and Treg monoclonal antibody (36), and immune checkpoint inhibitor monoclonal antibody (37); cellular immunotherapy such as chimeric antigen receptor T cell (CAR-T) immunotherapy (38) and chimeric antigen receptor natural killer (NK) cell (CAR-NK) (39, 40) immunotherapy and tumor vaccine immunotherapy (41–43). The combination of multiple immunotherapies is known to improve the efficacy, gradually making it an effective approach for future cancer treatment. However, immunotherapy has certain shortcomings (44). For example, the cell source of dendritic cell (DC) vaccine is limited and its treatment cost is high. Genetic vaccines are prone to degradation by nucleases *in vivo* and are associated with the risk of causing genetic mutations in normal cells. In addition, several malignant tumors can evade the surveillance mechanism of the immune system through a variety of ways. Therefore, a single immunotherapy method is insufficient to eradicate the tumor or provide long-term anti-tumor immune response. In this regard, combinations of multiple immunotherapies can improve effective and increase the long-term anti-tumor effect.

## 3 Potential of the Combination of Immunotherapy and Antiangiogenesis Therapy for Cancer

**Figure 2:**
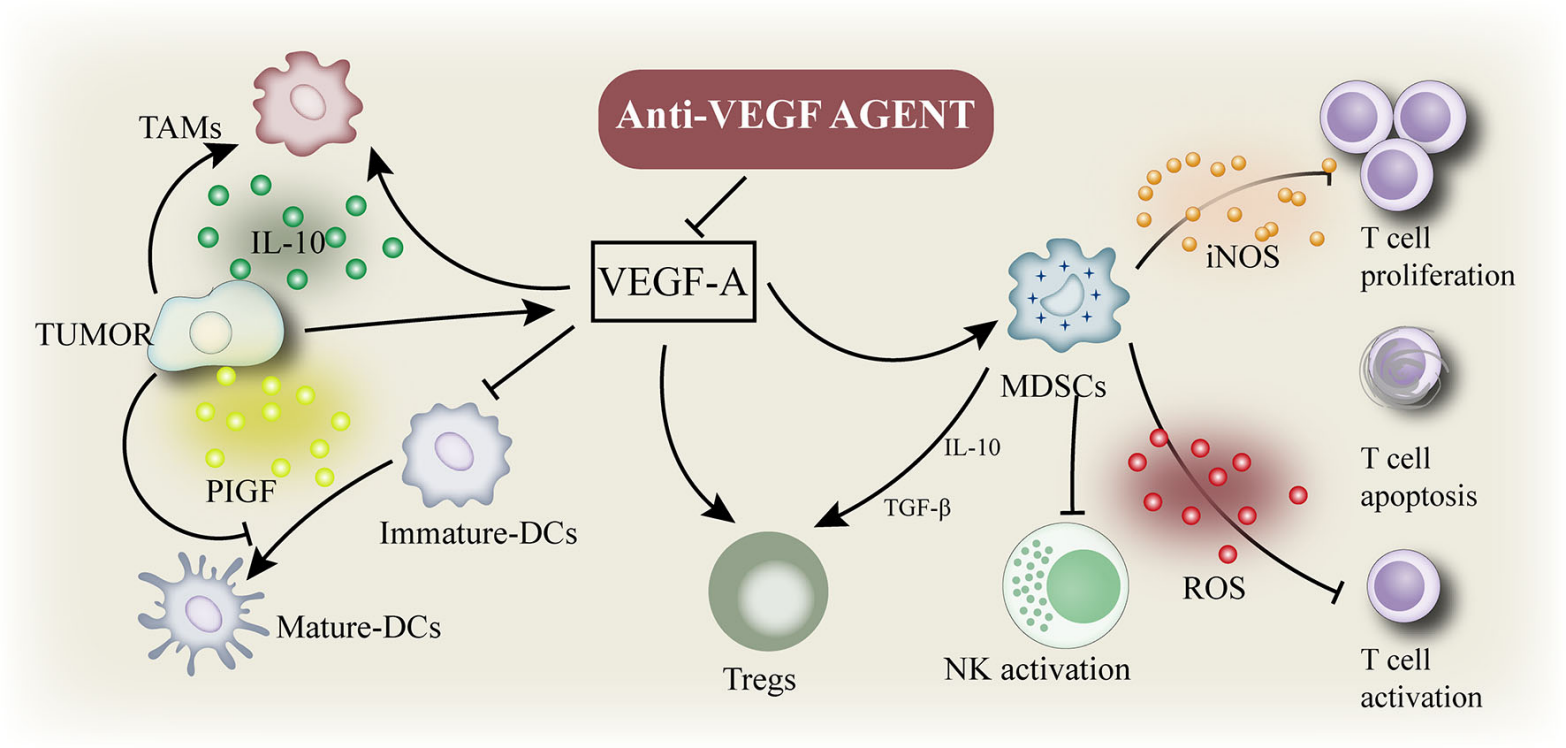
Immune suppressive microenvironment induced by VEGF: VEGF enhances the mobilization and proliferation of various cells, including regulatory T cells (Tregs), and the release of immunosuppressive cytokines. It also enhances the mobilization of tumor-associated macrophages (TAM) and their polarization to the M2 phenotype. VEGF also activates myeloid - derived suppressor cells (MDSCs), resulting in the release of more VEGF. In addition, VEGF inhibited dendritic cell maturation and antigen presentation during initiation. Thus, VEGF reduces the proliferation and activation of initial CD8+ cells by inhibiting the activity of dendritic cells even in the presence of neoantigens.

Tumor angiogenesis is a prominent marker of cancer cells (45). Angiogenesis stimulates tumor growth, progression, and metastasis by providing nutrition to tumor cells (46). The primary mediator of tumor angiogenesis is VEGF-A (47, 48), commonly known as VEGF, whose functions are exerted through the receptor tyrosine kinase VEGFR-2. VEGF inhibition increases the antigen presentation of DCs (49) and improves T cell migration from lymph nodes to the tumor site by normalizing the tumor vascular system (50). In addition, the numbers of Tregs, TAM, and MDSCs was inhibited (51) and the expression of immunosuppressive cytokines was negatively regulated. Therefore, VEGF inhibitors can reprogram the immunosuppressive TME into an immunostimulatory environment (**Figure 2**) (52). The application of PD-1/PD-L1 antibody can enhance the anti-tumor activity of T cells. As mentioned above, a combination of VEGF and PD-1/PD-L1 inhibitors can promote anti-tumor immunity. First, VEGF inhibition promotes the maturation of DCs, leading to an effective initiation and activation of T cells (53). Second, anti-VEGF antibody normalizes tumor vessels and promotes the effective recruitment of T cells to tumors (50). Third, anti-VEGF antibody inhibits MDSCs, Tregs, and TAMs, resulting in the reprogramming of immunosuppressive microenvironment into an immunostimulatory microenvironment (52). Fourth, PD-1/PD-L1 antibody can restore the ability of T cells to attack tumor cells. These four mechanisms can effectively inhibit tumor growth and provide cancer immunity. In addition, antiangiogenesis therapy treatment induces the formation of high endothelial venules (HEVs) (48), which promote T cell infiltration into solid tumors, thus improving the immune responses to cancer. These studies confirm that interventions targeting angiogenesis signaling in the TME can enhance the immunotherapy response (7).

## 4 Microbiome Influences Immunotherapy and Antiangiogenesis Therapy

### 4.1 The Role of Microbiota in Carcinogenesis

#### 4.1.1 Mechanism of Promoting Tumorigenesis by the Microbiome

Bacteria were first observed in tumors by Koch and Pasteur in the 19th century. In 1890, William Russell reported a cancer parasite. However, the concept that stated the presence of microorganisms inside tumors was refuted in the first half of the 20th century.

Nevertheless, growing evidence has led to the recognition that microorganisms can grow before a tumor originates and regulate the systemic responses to the tumor through their interactions with the immune system (25, 54, 55). For example, human cancer viruses can promote tumorigenesis by integrating oncogenes into the host genome. In addition, microorganisms can affect the stability of the genome, resulting in resistance to cell death and increased proliferation, which are known to drive the transformation. PKS^+^ *Escherichia coli* and colibactin-expressing *E. coli* can increase the probability of increase the probability of mice with intestinal tumor. Furthermore, studies support the role of enterotoxigenic *Bacteroides fragilis* in human and animal colon cancer models. Both *E. coli* and lethal cytotoxin (CDT) can cause double-stranded DNA damage in mammalian cells (56), whereas vulnerable bacteroidotoxin (BFT) acts indirectly by triggering the production of high levels of reactive oxygen species (ROS), thereby damaging the host DNA (57). Long- term high levels of ROS may exceed the host’s DNA repair mechanism, leading to DNA damage and mutations. In addition to destructive DNA, several microbial proteins (58) are known to be involved in the host carcinogenesis through the Wnt/β-catenin signaling pathway to regulate the stemness, polarity, and growth of cells. *B. fragilis* (59), which secretes enterotoxin, can stimulate the cleavage of E-cadherin by BTF, leading to the activation of β-catenin (60). *Salmonella typhimurium* strains can cause chronic infection by secreting a protein named Avra, which activates epithelial β-catenin (61) signaling and is associated with the development of hepatobiliary carcinoma (62, 63).

*Helicobacter pylori* is considered a carcinogenic pathogen and causes gastric atrophy and low hydrochloric acid. *Helicobacter*-induced gastric cancer is mediated by a complex interaction involving the microbiome. Bacterial overgrowth in the stomach subsequently increases the bacterial conversion of dietary nitrates to carcinogens (25). In contrast to promoting gastric cancer, patients infected with CagA^-^ positive *H. pylori* have a lower risk of esophageal adenocarcinoma than those infected with CagA^-^ *H. pylori* (64, 65). CagA^+^ *H. pylori* reduces the risk of human esophageal adenocarcinoma, which indicates the organ-specific role of the microbiome in carcinogenesis. In addition to gastric cancer, *H. pylori* can cause lung tumors (66). The lipopolysaccharide of *H. pylori* can induce the production of proinflammatory factors including interleukin (IL)-1, IL-6, and TNF1 that may induce chronic bronchitis, often accompanied by lung cancer (67). FADA, an adhesin produced by *C. nucleatum* promotes tumor development in CRC patients (68). It binds to E-cadherin and activates the Wnt/β-catenin signal transduction pathway to induce carcinogenesis. Certain microbiomes such as *Bacteroides* and *Burkholderia* are responsible for anti-tumor effects. In both cases, the growth of these bacterial species enhanced the T cell response.

#### 4.1.2 Mechanism of Inhibiting Tumorigenesis by the Microbiome

**Figure 3:**
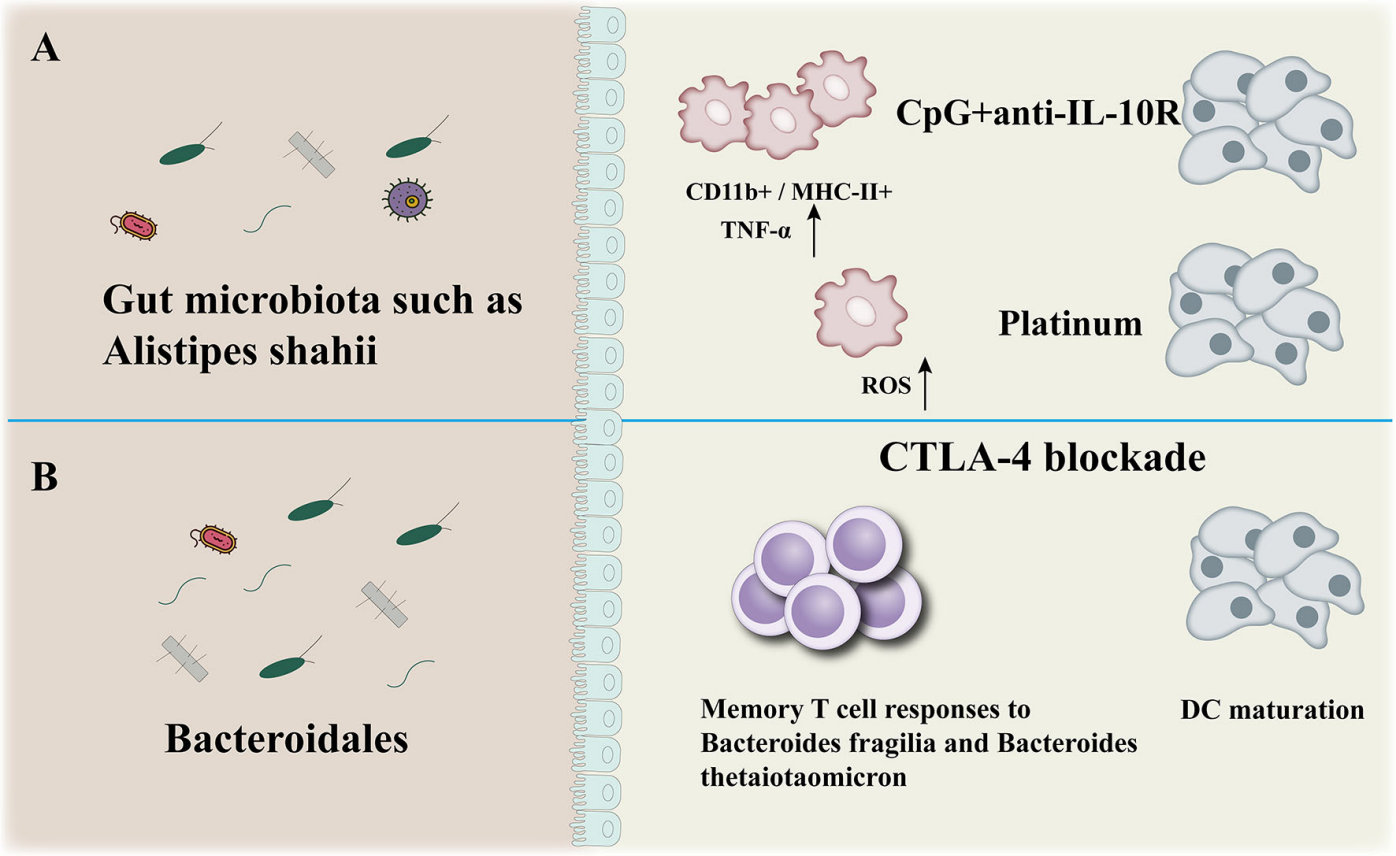
Anticancer effect of microbiota. The gut microbiota participates to the efficacy of immunotherapy (CpG+anti-IL-10, CTLA-4blockade) **(A, B)**: modulation of myeloid-derived cells production of TNF-a and reactive oxygen species (ROS) in mice **(A)**, enhancement of Bacteroidales-specific memory T cell responses in mice and patients and of DC maturation in mice **(B)**.

The gut microbiota participates to the efficacy of immunotherapy (CpG+anti-IL-10, CTLA-4blockade) (**Figure 3**). There is evidence that the wider community of commensal gut bacteria might modulate cancer risk and progression through competitive exclusion and other mechanisms (68). SCFAs (short­chain fatty acids), a microbial metabolites, produced during the colonic fermentation of otherwise indigestible carbohydrates (fibres or resistant starches), play a major role in maintaining intestinal homoeostasis and overall gut health. The SCFAs suppress the growth of Gram­negative pathogens, function as energy sources for colonocytes as well as other bacteria, dampen inflammation, and promote apoptosis of cancer cells (69). As such, bacteria involved in the biosynthesis and metabolism of short­chain fatty acids are actively involved in maintaining a stable and healthy gut community. Lower abundance of beneficial bacteria that produce short­chain fatty acids have been consistently observed across studies of colorectal cancer and experiments in murine models effectively show that dietary fibre protects against colorectal tumorigenesis in a microbiota­dependent and butyrate­dependent manner (70, 71).

Recently, more direct evidence in favor of the capacity of commensal bacteria to promote tumor immune surveillance was provided by comparing the growth of melanomas and their infiltration by IFN-γ producing CTLs in C57BL/6 mice from two different providers, Jackson Laboratories (JAX) and Taconic Farms (TAC) (72). TAC mice with poor immune surveillance exhibited a relative loss of *Bifidobacterium* species. Oral feeding of TAC mice with *Bifidobacterium* their cohousing with JAX mice restored defective processing and presentation of tumor antigens by dendritic cells (DCs), re-established infiltration of melanomas by CTLs and reduced malignant growth.

### 4.2 Microbiome Regulation as an Adjunct to Cancer Treatment

Several strategies to regulate the microbiota are being developed for a variety of human diseases, including cancer. These include the use of FMT (fecal microbiota transplantation), which is a safe and effective approved treatment for *C. difficile* relapse and is currently being experimentally used to treat inflammatory bowel a metabolite of bacteria disease (73), metabolic diseases (74) and cancer (75). In addition, other strategies for manipulating the microbiome are being studied for a variety of diseases, including probiotic administration. The term “probiotics” refers to a combination of bacteria or live bacteria that benefit the host’s health in an appropriate abundance. Another approach is to inject microbial consortium to improve human health since the early 20th century when Meichenikov won the Nobel Prize for his work in immunology based on the theory that administering microorganisms may exert beneficial effects. *Lactobacillus* and *Bifidobacterium* are the most commonly used probiotics in clinical practice (76). Other bacteria used as probiotics include *Saccharomyces boulardii* and *Bacillus* species. Probiotics affect the intestinal ecosystem by regulating the intestinal mucosal immunity, interacting with symbiotic bacteria or potentially harmful pathogens, producing metabolites such as SCFAs and bile acids. In addition, they act on host cells through signaling pathways and contribute to the suppression and elimination of potential pathogens, improve the intestinal microenvironment, strengthen the intestinal barrier, reduce inflammation, and enhance antigen-specific immune responses (77).

In the past few years, probiotics have been widely studied for their functions in humoral, cellular, and non-specific immune regulation, promoting immune barrier (78), increasing the production of peripheral immunoglobulin, stimulating IgA secretion, and reducing the production of pro-inflammatory cytokines. Recently, the International Association for Probiotics and Probiotics Science (ISAPP) has proposed a consensus definition, which defines prebiotics as “a substrate selectively used by host microorganisms endowed with health benefits” (79). GOS, a prebiotic consisting of galactose residues with glucose or galactose at the end, has been widely used in infant formula and other confectionery and beverages (80), as well as commercially available probiotics *Bifidobacterium* and *Lactobacillus* (81). *Akkermansia muciniphila* (AKK) is a kind of intestinal symbiotic bacterium that colonizes the mucosal layer and plays a crucial role in improving metabolic disorders (82) and immune response (83) of the host. Studies have shown that a combination of IL-2 and AKK exerts a strong anti-tumor effect on tumor tissues from colorectal cancer patients. Oral administration of AKK in subcutaneous melanoma and colorectal tumor-bearing mice enhanced the therapeutic effect of IL-2 significantly. The anti-tumor immune response initiated by AKK is partially mediated by Amuc_1100, which is derived from the outer membrane protein of AKK through the activation of the TLR2 signaling pathway (84). In addition, oral AKK supplementation protects the intestinal barrier function and maintains mucosal homeostasis in response to systemic IL-2 therapy (85). The abundance of *A. muciniphila* was increased about 100 times in genetically obese mice by administering prebiotic *fructooligosaccharides* (86). *Fructooligosaccharides* are fermented more rapidly and produce more butyric acid than inulin (87). In a simulated microbial environment (88), the addition of inulin increased the abundance of homogeneous or cocoa bacteria. Xylo-oligosaccharides (XOS) are produced from lignocellulosic materials rich in xylan. Studies have shown that certain probiotics such as *Bifidobacterium* and *Lactobacillus plantarum* use XOS more selectively due to the presence of xylanase system. Mannose oligosaccharides (MOS), oligomers composed of mannose residues, have been widely used to improve the growth and health performance of livestock and aquaculture. For example, the number of *C. perfringens* and *E. coli* decreased and the relative number of *Lactobacillus* increased when MOS was added to chicken feed (89).

### 4.3 Microbiome and Immunotherapy

The crosstalk between intestinal microbiota and the immune system is crucial both to develop tolerance to symbiotic bacteria and oral food antigens and prepare the immune system to recognize and attack opportunistic bacteria, thereby preventing bacterial invasion and infection (90). In addition to affecting the local immune response, the microbiota exerts an immense impact on innate and acquired immunity at multiple levels (91), as evident from the studies conducted using preclinical models (92). For example, sterile (GF) mice lacking intestinal microbiota developed severe immune deficiency, loss of mucus layer, changes in immunoglobulin A (IGA) secretion, and alterations in the size and function of Peyer’s patches and draining of mesenteric lymph nodes. Thus, microbes play a pivotal function to shape the immune system. Studies in GF or antibiotic-treated mice (93) have demonstrated impaired response to intratumoral injection of Toll-like receptor (TLR) agonists. The tumor-associated myeloid cells were activated by symbiotic intestinal bacteria *via* the TLR4 signaling to produce TNF and other inflammatory cytokines, which mediate the anti-tumor effects of these drugs. Bacteria have been implicated in the development of the stomach (63) (*H. pylori*) and colorectal cancer (94) (*Fusobacterium nucleatum*) by directly affecting the lumen mucosa through several different mechanisms.

#### 4.3.1 Effect of Gutmicrobiota Microbiota on Immune Checkpoint Blockade

**Figure 4:**
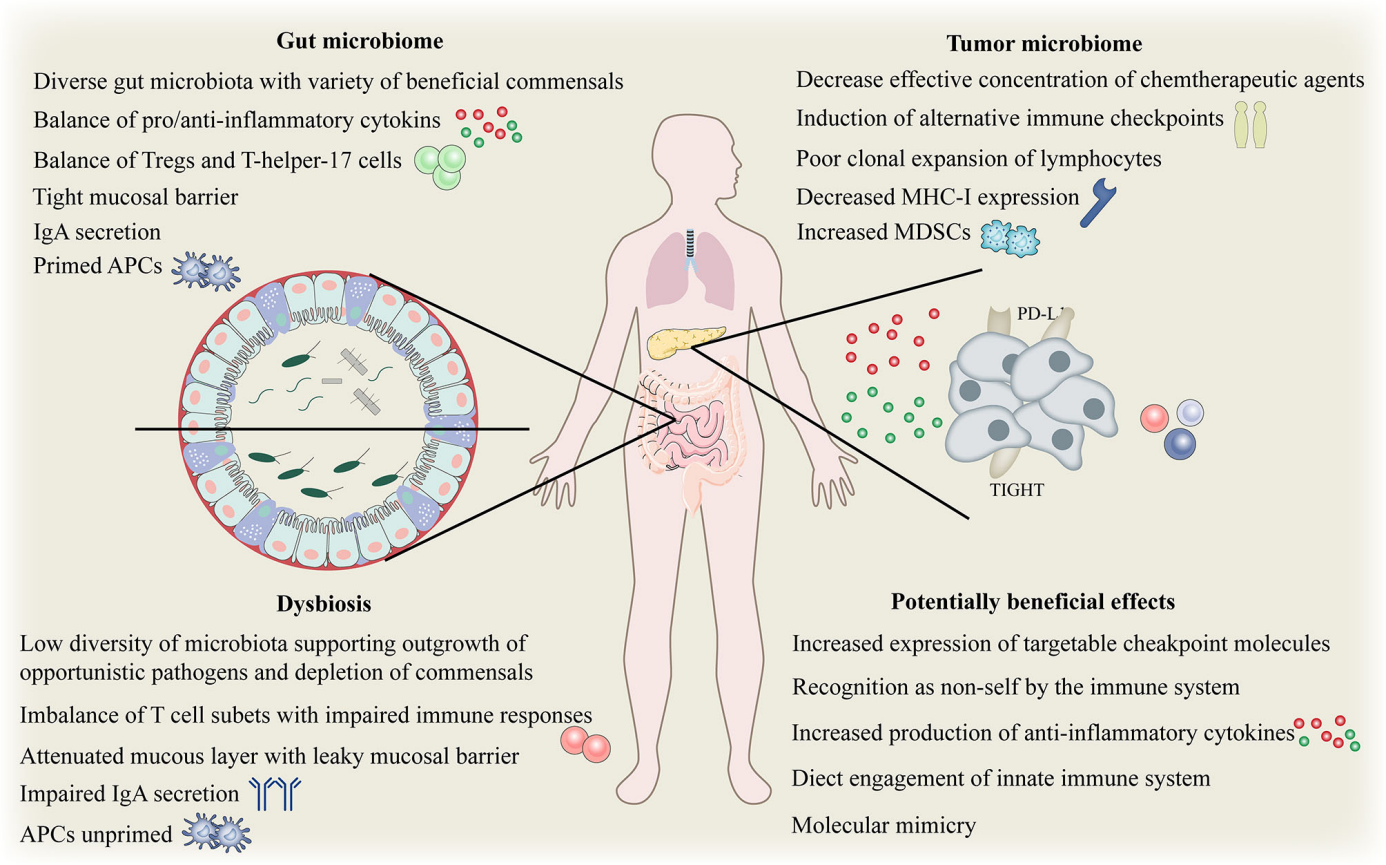
Complex interplay between gut and tumor microbiome and the host immune system. Complex interplay of the gut and tumor microbiome and the host immune system. APC, antigen-presenting cells; IgA, immunoglobulinA; MHC, major histocompatibility complex; Tregs, regulatory T cell; MDSCs, myeloid-derived suppressor cells; PD-L1, programmed death ligand 1.

Although the immune system has evolved to fight against invading pathogens, a delicate balance exists on the gut immune axis, that is, the tolerance to key symbiotic intestinal microbiota and food antigens, and the defense against intestinal pathogenic microbiota. Despite our incomplete understanding, there is growing evidence of numerous mechanisms which gut microbes may affect local and systemic immunity (72, 95–97). Gut microbiota is essential for maintaining the integrity of the mucosal barrier and preventing intestinal leakage. Intestinal leakage can allow the pathogenic or normal symbiotic bacteria to enter the bloodstream, activate pattern recognition receptors in distant locations, and trigger an immune response (98). (**Figure 4**). The gut microbiota and its metabolites, such as short-chain fatty acids (SCFAs) may disrupt the balance of anti-inflammatory and pro-inflammatory cytokines locally and systematically, thus disturbing the proportion of regulatory and helper T cell subsets (99). To effectively evade the infection, gut microbiota activates local phagocytes through tension signals (100). The effects of biological disorders on the immune function of the normal system include increased susceptibility to certain infections and altered response to vaccines (101). Increasing evidence suggests that biological disorders can affect both local and systemic anti-tumor immunity in a similar manner. For example, repeated exposure to antibiotics may be associated with an increased risk of cancer.

As part of the immune response, microbial-associated molecular patterns are responsible for providing “danger signals” that trigger the immune response. Gut microbiota determines the “tension” of immune response and thus plays an adjuvant role in immunotherapy (102). Three studies in *Science* support the idea that the composition of the gut microbiome modulates the response to immunotherapy in patients with epithelial tumors, including non-small cell lung (103) and kidney cancers (104) and melanoma (105) by blocking anti-PD-1 or PD-1 ligand 1(PD-L1). A study on non-small cell lung cancer showed that the biological dysregulation caused by broad-spectrum antibiotics was associated with the failure of targeted immunotherapy of PD-1 or PD-L1 in patients and mice. Fecal microbial transplantation (FMT) of sterile mice that responded to PD-1 therapy had enhanced anti-tumor immunity compared with mice that received FMT from unresponsive donors. The level of anti-tumor CD8+T cells was increased in mice receiving FMT from responders, whereas the level of immunosuppressive CD4+T cells was lower in mice receiving FMT from non-responders.

### 4.4 Microbiome and Antiangiogenesis Therapy

**Figure 5:**
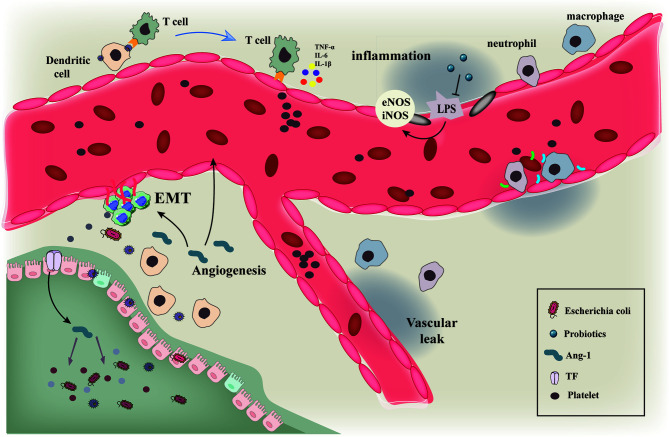
Schematic diagram showing the interaction of gut microbiota with immune cells and tumor vascular microenvironment. Schematic diagram of the interaction of gut microbiota and various microorganisms with immune cells and tumor vascular microenvironment.Intestinal microbiota and its secretions interact with immune cells such as macrophages and neutrophils to induce vascular remodeling and inhibit epithelial mesenchymal transition by regulating the processes of vascular inflammatory injury and hypoxia microenvironment.

Angiogenesis is one of the hallmarks of tumors and therefore one of the primary anti-cancer therapeutic approaches is to block angiogenesis, especially in solid tumors. Several bacterial strains have been reported to colonize the tumor environment and exert their oncolytic properties (**Figure 5**). Thus, a combination therapy using tumor-targeting bacteria along with anti-angiogenesis therapy can be effective in inhibiting angiogenesis and preventing tumor growth. The anti-tumor effect can be further enhanced by genetically engineering the bacteria to produce and secrete anti-angiogenic factors.

#### 4.4.1 Regulation of Tumor Angiogenesis by Microbial Metabolites

An array of studies has elucidated that metabolites secreted by microbiota can regulate tumor angiogenesis. Butyrate, an SCFA derived from the microbiota, participates in a series of cellular processes in a concentration-dependent manner. Low concentration sodium butyrate (NaBu) has been proved to promote angiogenesis (106). Nicotinic acid effectively resists iodoacetamide-induced colitis by improving pathological angiogenesis and inflammation in a GPR109A-dependent manner (107). A study demonstrated that a polypeptide of *E. coli* and its tripeptide analogs promoted tumor cell invasion and angiogenesis, thus potentially affecting tumor metastasis (108). Intestinal microbiota can selectively activate mucosal endothelial and mesenchymal cells to promote specific angiogenesis in a TLR- and NLR-dependent manner (109). This innate immune-mediated response may expand the mucosal microvascular network, promote the recruitment of immune cells, and lead to chronic intestinal inflammation. Remote ischemic preconditioning can prevent the increase in tumor burden caused by the congestion of the superior mesenteric vein and bacterial translocation due to portal vein occlusion by maintaining intestinal integrity and reducing bacterial translocation. Therefore, regulating liver exposure to enterogenous bacterial products is the key mechanism to mediate postoperative recurrence of hepatocellular carcinoma (110). Disturbance in intestinal microcirculation secondary to severe acute pancreatitis (SAP) can damage the intestinal mucosal barrier, intestinal microbiota translocation, and sepsis. The glycocalyx on vascular endothelium sustains its normal function by regulating the vascular permeability and inhibiting the intercellular adhesion. A study revealed that the glycocalyx degradation of endothelial cells during SAP was related to the damage of mesenteric microcirculation (111). In colitis, blood vessel epicardial substance (BVES) effectively limits the deterioration of mesenteric immune response and intestinal tissue damage caused by increased bacterial colonization and translocation. In addition, it is found that BVES is underexpressed in severe colitis (112).

#### 4.4.2 Influence of Intestinal Microbiota Structure on Tumor Antiangiogenesis Therapy

In addition to the metabolites secreted by the microbiota, the changes in the structure and composition of intestinal microbiota affect the tumor antiangiogenesis therapy. Certain studies have shown that a high-fat diet aggravates choroidal neovascularization (CNV) by changing the intestinal microbiota (113). Obesity is associated with intestinal disorders, usually manifested as mild inflammation, which subsequently affects vascular function. A high-fat diet in pregnant women can cause changes in the intestinal microbiota, impairing the intestinal barrier function, Furthermore, the placentas of obese pregnant women show vascular immaturity, hypoxia, increased levels of inflammatory transcription, autophagy, and changes in endoplasmic reticulum stress markers (114). Probiotics can significantly improve the intestinal barrier, reduce endothelial dysfunction and LPS-induced vascular oxidative stress, and decrease metabolic endotoxemia. In addition, it restores the increase in vascular superoxide levels in obese mice by reducing NADPH oxidase activity and increasing antioxidant enzymes (115). The intestinal microbiota can promote the interaction of intestinal mucosal cells with specific proteins to enhance signal transduction. Tissue factor (TF) is a kind of membrane receptor that activates the extrinsic coagulation pathway and promotes tumor growth and angiogenesis. Intestinal microbiota activates coagulation proteases and phosphorylates the TF cytoplasmic domain in the small intestine by localization of TF on the cell surface to effectively promote TF glycosylation. However, the anti-TF treatment decreased the intestinal vascular remodeling and Ang-1 expression induced by microbiota (116). Ang is a kind of antibacterial peptide secreted by the intestine; a change in Ang in feces has been reported in patients with inflammatory bowel disease. The expression of Ang1 in the intestinal microbiota of mice is induced by direct binding of α-Proteus destructs the integrity of the cell membrane to inhibit the growth α-Proteus, thus promoting the growth of Spirillum. The deletion of Ang-1 reduces the abundance of Trichospirillum α- Proteus increased, which aggravates DSS-induced colitis. Oral administration of Ang-1 is known to restore the intestinal microbiota composition of Ang-1-deficient mice, ultimately relieving colitis. Therefore, targeting Ang could be a potential therapeutic target for intestinal diseases associated with dysbacteriosis (117).

## 5 Discussion

The human microbiome comprises trillions of microbes that live on and within the human body. The symbiotic evolution of microbes within the human body is associated with several health benefits, including absorption of nutrients and maintenance of the integrity of the mucous membrane barrier. It is increasingly evident that the regulation of intestinal microbiota may represent a novel and important auxiliary means of current anti-cancer treatment. The regulation of probiotics and fecal bacteria transplantation indirectly regulate human health by affecting the microbial composition and structure of the body, which is a safe and effective method to treat recurrent *C. difficile* infection. The infinite potential of microbial regulation can be harnessed as adjuvant cancer immunotherapy. Numerous studies have suggested an interplay between traditional Chinese medicine (TCM) and gut microbiome to maintain a healthy host–microbiome holobiont status. Studies are being conducted to assess the physical interactions between the gut microbiome and the chemical composition of Chinese herbs. Studies have shown that obese mice treated with *Ganoderma* can lose weight by transplanting fecal bacteria. Similarly, Gegenqinlian decoction improves the blood glucose levels by altering the intestinal microbiota in patients with type 2 diabetes mellitus. Dajianzhong decoction (DJZT) can increase the abundance of *Clostridium* and *Lactococcus lactis*. Furtherexperiments show that DJZT can significantly improve the ability of fecal microorganisms to metabolize ginsenosides. Thus, TCM remodels the gut microbiota to enhance the overall health status and can play an efficient role in selectively promoting the growth of symbiotic probiotics. Similarly, polysaccharides extracted from Chinese herbs have been shown to exert prebiotic effects *in vitro*.

Being a very recent research field, there remain unsolved questions, in particular about the mechanism by which gut microbes interact with the human body and the exact bacterial species or groups of bacterial species that mediate anti-tumor effects. Thus, further studies are warranted in all areas ranging from basic research and translational research to clinical research and epidemiological analysis. A multifaceted strategy is required to monitor and adjust these factors to achieve optimal levels of health and effective treatment of cancer.

## Author Contributions

ZW and YL: conceptualization and design the review, and review final version approval. XW and MS: bibliographic research. XW, MS, and ZW: writing – original draft preparation. XW, MS, and YZ: table and figure design. AW and YZ: supervision. ZW and YL: funding acquisition. All authors contributed to the article and approved the submitted version.

## Funding

This work was supported by National Natural Science Foundation of China (82004124&81961128020), Natural Science Foundation of Jiangsu Province (BK20200154), Natural Science Foundation of Higher School of Jiangsu Province(18KJA360007) and the Jiangsu College graduate research and innovation projects under Grant (KYCX21_1757, SJCX21_0689).

## Conflict of Interest

The authors declare that the research was conducted in the absence of any commercial or financial relationships that could be construed as a potential conflict of interest.

## Publisher’s Note

All claims expressed in this article are solely those of the authors and do not necessarily represent those of their affiliated organizations, or those of the publisher, the editors and the reviewers. Any product that may be evaluated in this article, or claim that may be made by its manufacturer, is not guaranteed or endorsed by the publisher.
