# Corrigendum: Microbiome Crosstalk in Immunotherapy and Anti-Angiogenesis Therapy

**DOI:** 10.3389/fimmu.2022.887775

**Published:** 2022-04-21

**Authors:** Xueting Wan, Mengyao Song, Aiyun Wang, Yang Zhao, Zhonghong Wei, Yin Lu

**Affiliations:** ^1^Jiangsu Key Laboratory for Pharmacology and Safety Evaluation of Chinese Materia Medica, School of Pharmacy, Nanjing University of Chinese Medicine, Nanjing, China; ^2^Jiangsu Joint International Research Laboratory of Chinese Medicine and Regenerative Medicine, Nanjing, China; ^3^Jiangsu Collaborative Innovation Center of Traditional Chinese Medicine (TCM) Prevention and Treatment of Tumor, Nanjing University of Chinese Medicine, Nanjing, China; ^4^Department of Biochemistry and Molecular Biology, School of Medicine & Holistic Integrative Medicine, Nanjing University of Chinese Medicine, Nanjing, China

**Keywords:** microbiome, immunotherapy, immune checkpoint, angiogenesis, prebiotics, Chinese medicine

In the published article, there was an error in affiliation **3**. Instead of **“Key Laboratory of Drug Metabolism and Pharmacokinetics, State Key Laboratory of Natural Medicine, China Pharmaceutical University, Nanjing, China”**, it should be **“Jiangsu Collaborative Innovation Center of Traditional Chinese Medicine (TCM) Prevention and Treatment of Tumor, Nanjing University of Chinese Medicine, Nanjing, China”**.

The authors apologize for this error and state that this does not change the scientific conclusions of the article in any way. The original article has been updated.

## Publisher’s Note

All claims expressed in this article are solely those of the authors and do not necessarily represent those of their affiliated organizations, or those of the publisher, the editors and the reviewers. Any product that may be evaluated in this article, or claim that may be made by its manufacturer, is not guaranteed or endorsed by the publisher.

